# Post-acquisition CO_2_ Inhalation Enhances Fear Memory and Depends on ASIC1A

**DOI:** 10.3389/fnbeh.2021.767426

**Published:** 2021-10-29

**Authors:** Rebecca J. Taugher, Amanda M. Wunsch, Grace Z. Wang, Aubrey C. Chan, Brian J. Dlouhy, John A. Wemmie

**Affiliations:** ^1^Department of Psychiatry, University of Iowa, Iowa City, IA, United States; ^2^Department of Veterans Affairs Medical Center, Iowa City, IA, United States; ^3^Pappajohn Biomedical Institute, University of Iowa, Iowa City, IA, United States; ^4^Iowa Neuroscience Institute, University of Iowa, Iowa City, IA, United States; ^5^Department of Internal Medicine, University of Iowa, Iowa City, IA, United States; ^6^Department of Neurosurgery, University of Iowa, Iowa City, IA, United States; ^7^Department of Molecular Physiology and Biophysics, University of Iowa, Iowa City, IA, United States; ^8^Roy J. Carver Chair of Psychiatry and Neuroscience, University of Iowa, Iowa City, IA, United States

**Keywords:** carbon dioxide, pH, fear memory, acid-sensing ion channel, ASIC1a, novel object recognition (NOR)

## Abstract

A growing body of evidence suggests that memories of fearful events may be altered after initial acquisition or learning. Although much of this work has been done in rodents using Pavlovian fear conditioning, it may have important implications for fear memories in humans such as in post-traumatic stress disorder (PTSD). A recent study suggested that cued fear memories, made labile by memory retrieval, were made additionally labile and thus more vulnerable to subsequent modification when mice inhaled 10% carbon dioxide (CO_2_) during retrieval. In light of this finding, we hypothesized that 10% CO_2_ inhalation soon after fear acquisition might affect memory recall 24 h later. We found that both cue and context fear memory were increased by CO_2_ exposure after fear acquisition. The effect of CO_2_ was time-dependent, as CO_2_ inhalation administered 1 or 4 h after cued fear acquisition increased fear memory, whereas CO_2_ inhalation 4 h before or 24 h after cued fear acquisition did not increase fear memory. The ability of CO_2_ exposure following acquisition to enhance fear memory was not a general consequence of stress, as restraining mice after acquisition did not alter cued fear memory. The memory-enhancing action of CO_2_ may be relatively specific to fear conditioning as novel object recognition was impaired by post-training CO_2_ inhalation. To explore the molecular underpinnings of these effects, we tested if they depended on the acid-sensing ion channel-1a (ASIC1A), a proton-gated cation channel that mediates other effects of CO_2_, likely via its ability to sense acidosis induced during CO_2_ inhalation. We found that CO_2_ inhalation did not alter cued or context fear memory in *Asic1a^–/–^* mice, suggesting that this phenomenon critically depends on ASIC1A. These results suggest that brain acidosis around the time of a traumatic event may enhance memory of the trauma, and may thus constitute an important risk factor for developing PTSD. Moreover, preventing peritraumatic acidosis might reduce risk of PTSD.

## Introduction

Dysregulated fear memories can be extremely maladaptive, such as in post-traumatic stress disorder (PTSD; Kessler et al., 2017; Watson, 2019). Recent studies suggest that altering fear memories might be an effective treatment strategy (Nader, 2015; Kida, 2019; Uniyal et al., 2020). Much of this work has relied on rodent models of fear memory, such as Pavlovian fear conditioning in which an aversive unconditioned stimulus such as a foot shock is paired with a neutral conditioned stimulus such as a tone or context (Fenster et al., 2018). Several strategies have been employed to attenuate memory after fear conditioning. For example, inhibiting the conversion of a short-term memory to a long-term memory (Schafe and LeDoux, 2000; Duvarci et al., 2008) or developing an inhibitory extinction memory (Myers and Davis, 2007) reduces conditioned freezing. Others have decreased or erased memory by using retrieval to render the memory labile and inhibiting reconsolidation (Nader et al., 2000) or facilitating erasure via extinction (Monfils et al., 2009; Clem and Huganir, 2010).

A recent study found that if mice inhaled 10% carbon dioxide (CO_2_) during retrieval, cued fear memory was made more labile than with retrieval alone, making it more susceptible to modification via subsequent extinction or reconditioning. CO_2_ inhalation during cued retrieval facilitated insertion of Ca2 + -permeable AMPA receptors (CP-AMPARs), increased cAMP-response element binding protein (CREB) phosphorylation and re-activated a greater percentage of neurons in the memory trace as compared to retrieval alone (Du et al., 2017). In addition to being labile after a retrieval event, fear memories are labile for a period of time immediately following acquisition during which they are vulnerable to disruption (Schafe and LeDoux, 2000; Duvarci et al., 2008). Thus, we wondered if CO_2_ inhalation during this labile period following acquisition might similarly alter fear memory.

Moreover, Du et al. found that the CO_2_ effect on cued retrieval depended on the acid-sensing ion channel-1 (ASIC1A; Du et al., 2017). ASIC1A is a synaptic cation channel that is activated when extracellular pH drops (Waldmann et al., 1997; Wemmie et al., 2002, 2013; Zha et al., 2006). It is abundantly expressed in the brain, but is particularly abundant in fear circuit structures such as the amygdala (Wemmie et al., 2003; Coryell et al., 2007; Price et al., 2014), where it has been implicated in synaptic transmission and plasticity (Du et al., 2014; Chiang et al., 2015). Several CO_2_-induced behaviors depend on ASIC1A, likely because of its ability to sense CO_2_-induced acidosis, including acid- and CO_2_-evoked freezing, CO_2_ aversion, CO_2_ conditioned place avoidance, and CO_2_-enhanced center avoidance (Ziemann et al., 2009; Taugher et al., 2014). Therefore, we hypothesized that any effects of post-acquisition CO_2_ inhalation would critically depend on ASIC1A.

## Materials and Methods

### Mice

*Asic1a^–/–^* mice, in which *Asic1a* but not *Asic1b* transcription is disrupted, were generated as previously described (Wemmie et al., 2002). Mice were maintained on a congenic C57BL/6J background. Mice had *ad libitum* access to water and chow (Teklab) and all mice were group housed. Mice were kept on a 12 h light/dark cycle; all experiments were conducted during the light phase. Both male and female mice were used in these studies and experimental groups were sex- and age-matched (10–20 weeks of age). Separate groups of mice were used for each experiment. All experiments were approved by the University of Iowa Animal Care and Use Committee and animal care met National Institutes of Health Standards.

### Cued Fear Conditioning

On the acquisition day, mice were placed in a near-infrared video fear conditioning chamber (Med Associates, Inc.) for a total of 14 min. During the first 3 min mice explored the chamber, and then a series of 5 tones (3 kHz, 80 dB, 20 s) were played each co-terminating with a shock (0.75 mA, 1 s) administered via the floor rods. There was a 120 s intertrial interval between tone/shock presentations. 24 h later, cue-evoked responses were assessed in a novel context in which lighting, odor, and floor texture had been altered. Mice were in this context for a total of 10 min with the tone continuously presented during minutes 4–6. A no-shock control group was performed with identical methods as described above except that foot shocks were omitted. VideoFreeze software (Med Associates, Inc.) was used to quantify freezing during acquisition and testing. Freezing is quantified during the entirety of acquisition and during the time that the tone is played during the cued test, with freezing normalized to the air-treated condition. Freezing in seconds (mean ± SEM) for each group is reported in Supplementary Table 1.

### Gas Exposure

Mice were exposed to 10% CO_2_ or air in an airtight clear Plexiglas container (20.3 cm × 20.3 cm × 16.5 cm) into which gas was infused at a rate of 5 L/min for 30 min. In order to avoid context generalization, gases were administered in a different room than fear conditioning and different cleaning products were used in the fear conditioning and gas contexts to give the chambers distinct odors.

### Context Fear Conditioning

On the acquisition day, mice were placed in a near-infrared video fear conditioning chamber (Med Associates, Inc.) for a total of 8 min. During the first 3 min mice explored the chamber, and then a series of 5 shocks (0.75 mA, 1 s) were administered via the floor rods with a 60 s intertrial interval. 24 h later, mice were returned to the acquisition context for 6 min in the absence of foot shocks. As with cued conditioning, freezing was quantified during acquisition and testing using VideoFreeze software (Med Associates, Inc.) and normalized to the freezing in the air-treated condition. Freezing in seconds (mean ± SEM) for each group is reported in Supplementary Table 1.

### Restraint Stress

Mice were placed in a plastic restraint tube fashioned from a 50 mL conical tube for 30 min. Non-stressed control mice remained in the home cage during this time.

### Novel Object Recognition

On days 1–3, mice were habituated to the open field apparatus (40.6 cm × 40.6 cm × 36.8 cm; ViewPoint) for 30 min per day. On the day 4, mice underwent an object recognition acquisition session in which they explored two identical objects (inverted beaker or media bottle placed in the back left and front right corners) in the open field apparatus for 15 min. On day 5, novel object recognition (NOR) was tested by replacing one of the familiar objects with a novel object (inverted beaker or media bottle) and allowing the mice to explore both objects in the open field for 10 min. The order in which objects were presented was counterbalanced. Mice were videotaped during acquisition (day 4) the object recognition test (day 5) and an experimenter blinded to genotype and condition assessed the amount of time the animal spent exploring each object (defined as having been within 2 cm of the object and their nose oriented toward the object). The discrimination ratio on the acquisition day was calculated by dividing the time spent exploring the left object by the total time spent exploring either object, and on the test day was calculated by dividing the time spent exploring the novel object by the total time spent exploring either object.

### Statistical Analysis

**FIGURE 1:**
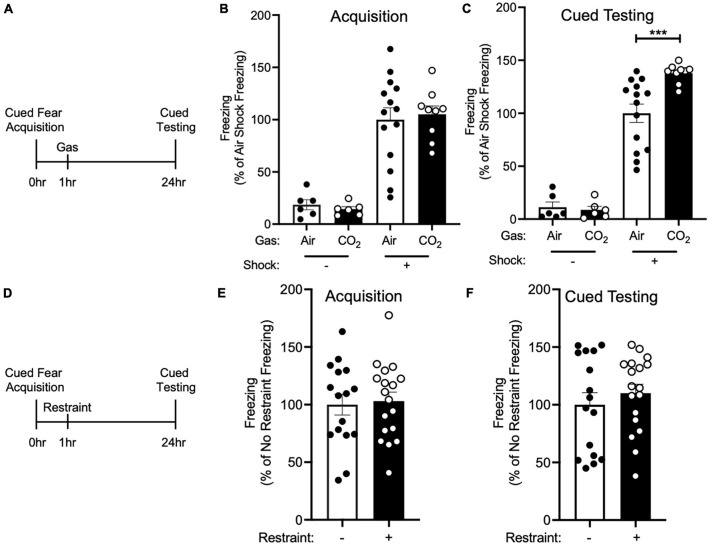
CO_2_ Inhalation after cued fear conditioning enhances fear memory. **(A)** Experimental timeline. 1 h after cued fear acquisition, mice were exposed to air or 10% CO_2_ for 30 min. 24 h after acquisition, cued fear memory was assessed. **(B)** During acquisition, much less freezing was observed when foot shocks were omitted from the training protocol [effect of shock, two-way ANOVA *F*(1, 31) = 61.22, *p* < 0.0001, *n* = 6–14]. Freezing in mice assigned to Air and CO_2_ treatment was similar in both the no shock (*p* = 0.8135) and shock groups (*p* = 0.6957). **(C)** On the cued fear test day, robust freezing to the tone was observed in mice who had received foot shocks during acquisition and this freezing was enhanced by CO_2_ treatment [gas by shock interaction, two-way ANOVA *F*(1,31) = 6.389, *p* = 0.0168]. Planned contrast testing revealed a robust effect of CO_2_ treatment in mice that had received foot shocks during acquisition (***p = 0.0004), whereas in contrast, CO_2_ treatment had no effect on freezing in the no shock control group (*p* = 0.8453). **(D)** Experimental timeline. 1 h after cued fear acquisition, mice were placed in a restraint tube for 30 min or left in the home cage. 24 h after acquisition, cued fear memory was assessed. **(E)** Freezing during acquisition was similar in both treatment groups [*t*(32) = 0.2560, *p* = 0.7996]. **(F)** Freezing during cued fear testing was not altered by restraint stress [*t*(32) = 0.7850, *p* = 0.4382].

**FIGURE 2:**
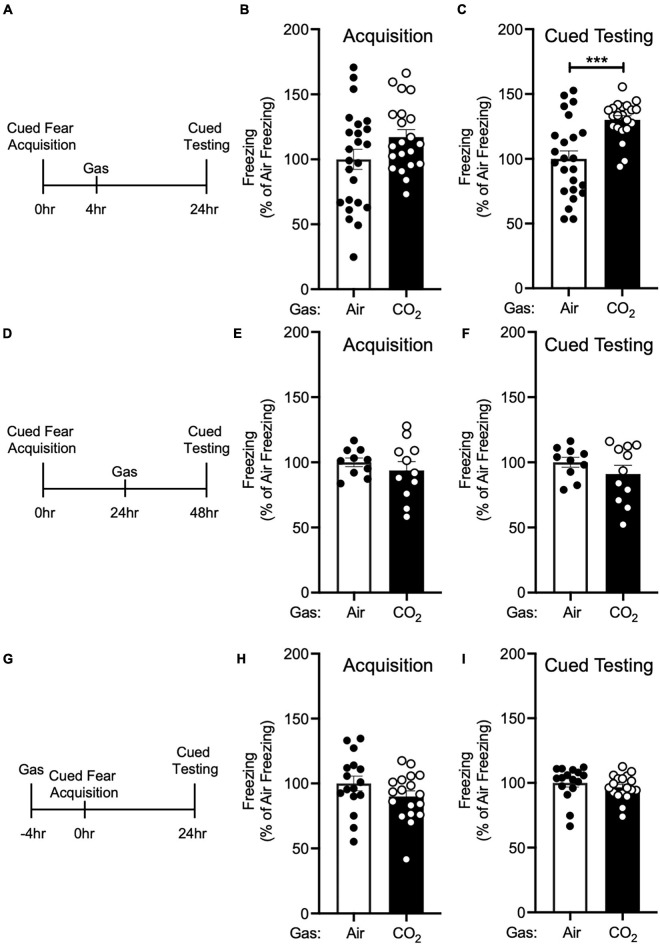
The enhancing effect of CO_2_ inhalation on cued fear memory is time-dependent. **(A)** Experimental timeline. 4 h after cued fear acquisition, mice were exposed to air or 10% CO_2_ for 30 min. 24 h after acquisition, cued fear memory was assessed. **(B)** Freezing during acquisition prior to gas exposure did not differ significantly between air and 10% CO_2_-treated groups [*t*(43) = 1.748, *p* = 0.0876, *n* = 24, 21]. **(C)** Exposure to 10% CO_2_ 4 h after acquisition increased freezing during the cued fear test [*t*(34.49) = 4.359, ***p = 0.0001]. **(D)** Experimental timeline. 24 h after cued fear acquisition, mice were exposed to air or 10% CO_2_ for 30 min. 24 h after gas exposure, cued fear memory was assessed. **(E,F)** In mice exposed to gas 24 h after acquisition, freezing during acquisition prior to gas exposure did not differ between air and 10% CO_2_-treated groups **(E)** [*t*(14.49) = 0.8350, *p* = 0.4173, *n* = 10, 11] nor did freezing during the cued fear test **(F)** [*t*(19) = 1.129, *p* = 0.2731]. **(G)** Experimental timeline. 4 h before cued fear acquisition, mice were exposed to air or 10% CO_2_ for 30 min. 24 h after acquisition, cued fear memory was assessed. **(H,I)** When acquisition occurred 4 h after gas exposure, CO_2_ treatment had no effect on freezing during acquisition **(H)** [*t*(32) = 1.435, *p* = 0.1610, *n* = 16, 18] or freezing during the cued fear test was observed **(I)** [*t*(32) = 0.8642, *p* = 0.3939].

An unpaired Student’s *t*-test was used to test for statistical significance between 2 groups. Welch’s correction was applied when an *F* test revealed a significant difference in variance between groups. A one sample *t* test was used to test if the mean of a group differed from random chance. A two-way ANOVA was used to test for statistical significance where a 2 × 2 experimental design was used. Outliers were identified using ROUT (Motulsky and Brown, 2006), *Q* = 1%. 3 outliers were removed from the 10% CO_2_ group in Figures 2B,C, 1 outlier was removed from the air group in Figures 2E,F, 2 outliers were removed from the air group and 1 outlier was removed from the 10% CO_2_ group in Figures 4E,F, and 1 outlier was removed from the air group in Figures 4H,I. *p* < 0.05 was considered significant. All statistical analyses were performed in Graphpad Prism.

**FIGURE 3:**
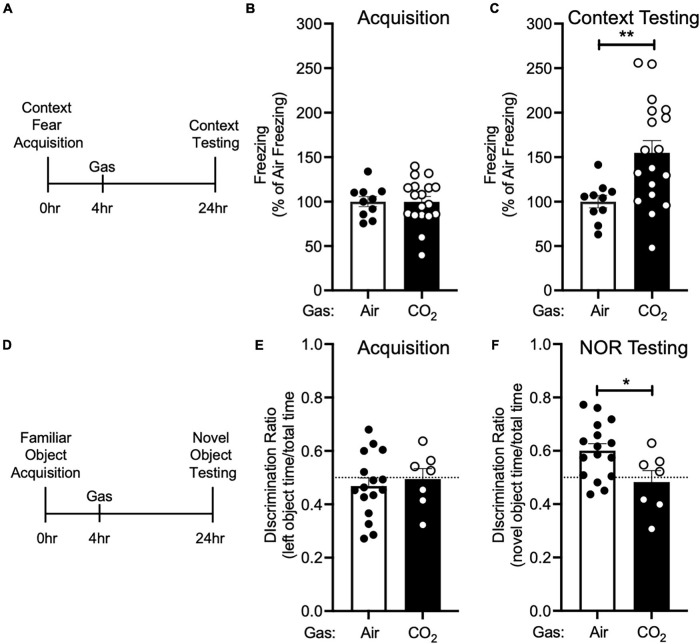
CO_2_ Inhalation after acquisition enhances context fear memory, but decreases novel object recognition **(A)** Experimental timeline. 4 h after context fear acquisition, mice were exposed to air or 10% CO_2_ for 30 min. 24 h after acquisition, context fear memory was assessed. **(B)** Freezing during acquisition prior to gas exposure did not differ between air and 10% CO_2_-treated groups [*t*(26) = 0.01532, *p* = 0.9879, *n* = 10, 18]. **(C)** Exposure to 10% CO_2_ 4 h after acquisition increased freezing during the context fear test [*t*(23.95) = 3.531, **p = 0.0017]. **(D)** Experimental timeline. 4 h after novel object acquisition, mice were exposed to air or 10% CO_2_ for 30 min. **(E)** Prior to gas exposure, object preference during acquisition did not differ between air and 10% CO_2_-treated groups [*t*(21) = 0.4902, *p* = 0.6290, *n* = 16, 7] and neither air [*t*(15) = 1.050, *p* = 0.3104] nor 10% CO_2_-treated [*t*(6) = 0.1454, *p* = 0.8891] groups preferred the left or right objects. **(F)** Exposure to 10% CO_2_ 4 h after acquisition decreased discrimination between the novel and familiar objects [*t*(21) = 2.409, *p = 0.0253]. Novel object preference was observed in the air group [*t*(15) = 3.805, *p* = 0.0017], but not the 10% CO_2_-treated group [*t*(6) = 0.7037, *p* = 0.7037].

**FIGURE 4:**
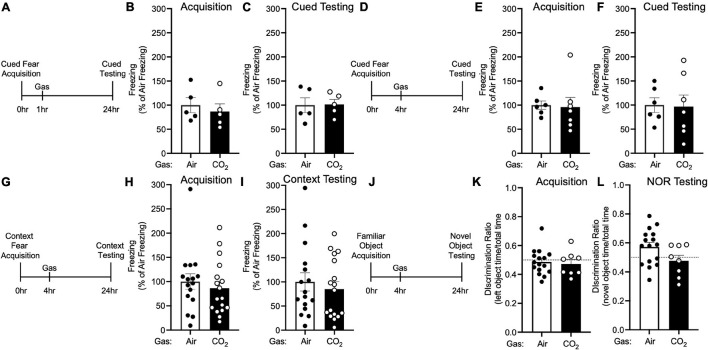
The effects of CO_2_ Inhalation on fear memory depend on ASIC1A. **(A)** Experimental timeline. 1 h after cued fear acquisition, *Asic1a^–/–^* mice were exposed to air or 10% CO_2_ for 30 min. 24 h after acquisition, cued fear memory was assessed. **(B,C)** Prior to gas exposure, freezing during acquisition prior to gas exposure did not differ between air and 10% CO_2_-treated groups **(B)** [*t*(8) = 0.5867, *p* = 0.5736, *n* = 5/group] nor did freezing after gas exposure during the cued fear test **(C)** [*t*(8) = 0.07644, *p* = 0.9409]. **(D)** Experimental timeline. 4 h after cued fear acquisition, mice were exposed to air or 10% CO_2_ for 30 min. 24 h after acquisition, cued fear memory was assessed. **(E,F)** Freezing during acquisition prior to gas exposure did not differ between air and 10% CO_2_-treated groups **(E)** [*t*(11) = 0.1697, *p* = 0.8683, *n* = 6, 7] nor did freezing during the cued fear test **(F)** [*t*(11) = 0.1077, *p* = 0.9162]. **(G)** Experimental timeline. 4 h after context fear acquisition, mice were exposed to air or 10% CO_2_ for 30 min. 24 h after acquisition, context fear memory was assessed. **(H,I)** Freezing during acquisition did not differ between air and 10% CO_2_-treated groups **(H)** [*t*(31) = 0.6410, *p* = 0.5262, *n* = 16, 17] nor did freezing during the context fear test **(I)** [*t*(31) = 0.5469, *p* = 0.6091]. **(J)** Experimental timeline. 4 h after novel object acquisition, mice were exposed to air or 10% CO_2_ for 30 min. **(K)** Prior to gas exposure, object preference during acquisition prior to gas exposure did not differ between air and 10% CO_2_-treated groups [*t*(22) = 0.3543, *p* = 0.7265, *n* = 16, 8] and neither air [*t*(15) = 0.6143, *p* = 0.5482] nor 10% CO_2_-treated [*t*(7) = 0.8899, *p* = 0.4031] groups preferred the left or right objects. **(L)** A trend toward a reduction in discrimination between the novel and familiar objects was observed in 10% CO_2_-treated mice [*t*(22) = 1.878, *p* = 0.0737]. Novel object preference was observed in the air group [*t*(15) = 2.367, *p* = 0.0318], but not the 10% CO_2_-treated group [*t*(7) = 0.6167, *p* = 0.5570].

## Results

Because inhaling 10% CO_2_ during the labile period induced by cued fear retrieval further enhanced memory lability (Du et al., 2017), we hypothesized that exposing mice to 10% CO_2_ 1 h after acquisition, during the time period in which new memories are thought to be labile (Mednick et al., 2011), would also alter fear memory. Thus, we trained mice in a cued fear conditioning paradigm in which we paired an auditory cue with a foot shock and 1 h later mice were placed in a different context and exposed mice to air or 10% CO_2_. 24 h after acquisition, mice underwent cued testing in which they were presented with the auditory tone in a novel context in the absence of foot shocks and conditioned freezing was assessed (Figure 1A). Prior to gas exposure, the air and 10% CO_2_-treated groups exhibited similar levels of freezing during cued fear acquisition (Figure 1B). Interestingly, the mice that had been exposed to 10% CO_2_ displayed a marked increase in freezing during cued testing as compared to air-exposed controls (Figure 1C). This suggests that CO_2_ exposure during the time period following acquisition can potentiate fear memory.

To rule out the possibility that CO_2_ exposure might have an effect on freezing that was independent of cued fear conditioning, we performed a no shock control experiment. The same cued fear acquisition paradigm (Figure 1A) was used, except that foot shocks were omitted from the protocol. This resulted in minimal freezing during both acquisition and cued fear testing, with 10% CO_2_ treatment having no effect on freezing (Figures 1B,C). This suggests that the effect of CO_2_ on cued fear memory depends on cued fear conditioning, rather than being a non-specific effect of CO_2_ exposure.

Because exposure to 10% CO_2_ is aversive and may induce stress (Ziemann et al., 2009; Taugher et al., 2014; Spiacci et al., 2018), we next sought to test if exposing mice to a different stressor would recapitulate the effects of CO_2_ inhalation on cued fear memory. We chose restraint stress given its widespread use as a method for inducing stress in mice (Stewart et al., 2008; Roper et al., 2010). We trained mice in a cued fear conditioning paradigm and 1 h later mice were either placed in a restraint tube for 30 min or left in the home cage (Figure 1D). Both treatment groups displayed a similar level of freezing during acquisition (Figure 1E), and restraint stress did not alter cued fear memory (Figure 1F). This suggests that the effects of 10% CO_2_ exposure might not be the result of its ability to induce stress, but instead the result of its other attributes, such as its ability to induce acidosis.

Next, we sought to determine if this effect of 10% CO_2_ on cued fear memory was time-dependent. First, we administered air or 10% CO_2_ 4 h after cued fear conditioning (Figure 2A). Prior to gas exposure, the air and 10% CO_2_-treated groups exhibited similar levels of freezing during acquisition (Figure 2B). Mice treated with CO_2_ 4 h after acquisition displayed much more freezing during cued testing than air-treated controls (Figure 2C), similar to when CO_2_ was given 1 h after acquisition (Figure 1C). To determine if this effect would extend to later time points, we administered air or 10% CO_2_ 24 h after cued fear conditioning and tested cued fear memory 48 h after acquisition (Figure 2D). Prior to gas exposure, the air and 10% CO_2_-treated groups exhibited similar levels of freezing during acquisition (Figure 2E). No effect of gas exposure was seen during cued testing (Figure 2F), suggesting that there is a critical time period after acquisition for 10% CO_2_ inhalation to affect cued fear memory. Next, we sought to determine if CO_2_ exposure prior to acquisition would have a similar effect on cued fear memory as CO_2_ exposure after acquisition. Thus, we administered air or 10% CO_2_ 4 h before cued fear conditioning and assessed cued fear memory 24 h later (Figure 2G). 10% CO_2_ pre-treatment did not affect freezing during acquisition (Figure 2H) or cued testing (Figure 2I), suggesting that it might be necessary for 10% CO_2_ exposure to occur after acquisition in order to potentiate fear memory.

We next sought to determine if the effect CO_2_ on fear memory was specific to cued fear conditioning. Thus, we performed context fear conditioning, a form of Pavlovian conditioning in which an association is made between a neutral context and a series of foot shocks. 4 h after context fear acquisition, we administered air or 10% CO_2_, and 24 h after acquisition, we returned mice to the acquisition context and assessed conditioned freezing responses (Figure 3A). Prior to gas exposure, the air and 10% CO_2_-treated groups exhibited similar levels of freezing during acquisition (Figure 3B). Similar to cued fear conditioning (Figure 2C), mice treated with 10% CO_2_ 4 h after context fear acquisition exhibited more freezing during context testing than air-treated controls (Figure 3C). This suggests that CO_2_ following acquisition has similar effects on cued and context fear memory.

To test if CO_2_ could also potentiate other types of memory, we tested its effects on NOR. Mice acquired a memory of the familiar object by exploring two identical objects. 4 h later, we exposed mice to air or 10% CO_2_. 24 h after acquisition, we assessed NOR by replacing one of the familiar objects with a novel object and assessing the amount of time that mice spent interacting with the novel and familiar objects (Figure 3D). During acquisition, mice exhibited similar levels of interaction with each object, and no difference between treatment groups was observed (Figure 3E). Surprisingly, when NOR was assessed 24 h later, we found that 10% CO_2_ treatment had decreased NOR as compared to air-treated controls and also caused a failure to discriminate between the novel and familiar objects (Figure 3F). This suggests that CO_2_ may affect different types of memory in distinct ways.

To explore the molecular underpinnings of these effects, we tested if they depended on ASIC1A. ASIC1A is a proton-gated cation channel that is known to mediate other effects of CO_2_, likely via its ability to sense acidosis induced by CO_2_ inhalation (Ziemann et al., 2009; Taugher et al., 2014). Thus, we tested the effects of 10% CO_2_ exposure on memory in *Asic1a^–/–^* mice under the same conditions in which CO_2_ enhanced fear memory in wild-type mice. Neither administering 10% CO_2_ 1 h after (Figures 4A–C) nor 4 h after (Figures 4D–F) cued fear acquisition altered freezing during cued testing in *Asic1a^–/–^* mice. Similarly, we found no effect of 10% CO_2_ administered 4 h after context fear acquisition on freezing in *Asic1a^–/–^* mice during context testing (Figures 4G–I). Together, these observations suggest that ASIC1A is critical for the effects of CO_2_ exposure on fear memory observed in wild-type mice.

Next, we tested whether the reduction in NOR induced by CO_2_ exposure similarly depended on ASIC1A (Figure 4J). Prior to gas exposure, *Asic1a^–/–^* mice exhibited similar levels of interaction with each object (Figure 4K). When gas was administered 4 h after acquisition, novel object preference was intact in air-treated *Asic1a^–/–^* mice, but not in those exposed to 10% CO_2_ (Figure 4L). However, the difference between air versus CO_2_-exposed *Asic1a^–/–^* mice did not reach significance (*p* = 0.07). These results are similar to those seen in wild-type mice, and suggest that in contrast to fear memory, the effects of CO_2_ exposure on NOR may be independent of ASIC1A.

## Discussion

Our results identify a novel effect of post-acquisition CO_2_ exposure on both cued and context fear memory. We found that during a discrete time period, including 1 and 4 h after fear acquisition, but not 24 h after, 10% CO_2_ inhalation increased fear memory. In contrast, there was no effect of CO_2_ exposure under the same conditions in *Asic1a^–/–^* mice, suggesting that ASIC1A is critical for this effect. Moreover, CO_2_ may affect distinct types of memory in different ways, as in contrast to these enhancements in fear memory, NOR was decreased by post-training CO_2_ inhalation.

These observations raise questions about where and how CO_2_ and ASIC1A act to produce their effects on fear memory. The amygdala is a likely site of ASIC1A action. ASIC1A is abundantly expressed in the amygdala (Wemmie et al., 2003; Coryell et al., 2007; Price et al., 2014; Chiang et al., 2015), and manipulating ASIC1A in the amygdala alters cued and context fear memory (Coryell et al., 2008; Chiang et al., 2015), as well as CO_2_-evoked freezing (Ziemann et al., 2009). Likewise, localized acidosis in the amygdala promotes freezing (Ziemann et al., 2009) and plasticity (Du et al., 2017). However, ASIC1A is also expressed in several other fear circuit structures in which it could influence fear memory, such as the bed nucleus of the stria terminalis, periaqueductal gray, hippocampus, prefrontal, and cingulate cortices (Wemmie et al., 2003; Coryell et al., 2007; Price et al., 2014). On the cellular level, these effects are likely due to ASIC1A in neurons. Neurons are a critical site of ASIC1A action in cued and context fear memory and CO_2_-induced freezing (Taugher et al., 2017), though it is possible that ASIC1A in non-neuronal cells could also be involved.

The acidosis caused by CO_2_ inhalation may activate ASIC1A directly (Ziemann et al., 2009). CO_2_ may also influence the activation of ASIC1A by protons released during neurotransmission (Du et al., 2014; Kreple et al., 2014; Gonzalez-Inchauspe et al., 2017). Either or both of these mechanisms might contribute to the effects of CO_2_ and ASIC1A observed here. Interestingly, acidosis not only activates these channels, it also inhibits them through inactivation and steady-state desensitization (Waldmann et al., 1997; Sherwood et al., 2012). These inhibitory effects of acid exposure have been demonstrated *in vitro*, although it is not clear to what degree they occur *in vivo*. Steady-state desensitization can be limited by endogenous peptides such as dynorphins and RF-amides (Sherwood and Askwith, 2008, 2009), raising the possibility that densitization *in vivo* may be less pronounced. It is also conceivable that the ASIC1A effects observed here may not require sustained channel activity.

The mechanisms downstream of ASIC1A are not yet clear. We speculate that the effects of CO_2_ and ASIC1A on fear memory may depend on CP-AMPARs in the postsynaptic membrane of lateral amygdala neurons, and or CREB-mediated transcriptional regulation. Conversion of short term fear memory to long lasting memory (i.e., consolidation) has been previously found to require both CP-AMPAR insertion (Rumpel et al., 2005; Nedelescu et al., 2010), and CREB phosphorylation and subsequent changes in gene transcription (Alberini, 2009). Previously, it was shown that when coupled with memory reactivation, CO_2_ exposure also increased both insertion of CP-AMPARs and CREB phosphorylation, which may be critical for promoting susceptibility of the fear memory to subsequent modification (Du et al., 2017). The potential role of ASIC1A in CREB phosphorylation has not been tested. However, the increase in CP-AMPAR insertion critically depended on the presence of ASIC1A (Du et al., 2017). Thus, CP-AMPAR insertion and CREB dependent transcription would seem good candidates for mediating the fear memory effects of CO_2_ and ASIC1A reported here.

Besides potentiating fear memory, our results suggests that CO_2_ may decrease other types of memory. 10% CO_2_ administered 4 h after acquisition attenuated NOR memory (Figure 4F). This may reflect the partially overlapping circuitry of these behaviors (Kim and Jung, 2006; Warburton and Brown, 2015; Fenster et al., 2018) or distinct molecular mechanisms being at play. Interestingly, whereas 10% CO_2_ had no effect on cued and context fear memory in *Asic1a^–/–^* mice, there was a strong trend toward a decrease in NOR in *Asic1a^–/–^* mice, suggesting that ASIC1A might mediate the fear conditioning, but not object recognition memory. Although additional work will be needed to identify the pH-sensitive molecules involved in the CO_2_ effect on novel object recognition, there are numerous pH-sensitive ion channels and receptors at synapses that could potentially be involved (Wemmie, 2011). Additional investigation will be needed to explore the effects of CO_2_ inhalation on other types of memory and other receptors.

Together, these observations suggest that brain pH around the time of a traumatic event may impact the associated fear memory, potentially altering the risk of developing PTSD. Consistent with this others have observed elevated levels of PTSD in patients with conditions that may result in a chronic respiratory acidosis such as asthma (O’Toole and Catts, 2008; Allgire et al., 2021), chronic obstructive pulmonary disease (Teixeira et al., 2015), obstructive sleep apnea (Colvonen et al., 2015), COVID-19 (Mertz Schou et al., 2021), and patients who are mechanically ventilated for a prolonged period of time (Wade et al., 2013). This suggests that pH alterations such as respiratory or metabolic acidosis or changes in acid-base equilibrium might contribute to PTSD risk and raises the exciting possibility that preventing acidosis or inducing alkalosis after a traumatic event could attenuate the associated fear memory.

## Data Availability Statement

The raw data supporting the conclusions of this article will be made available by the authors, without undue reservation.

## Ethics Statement

The animal study was reviewed and approved by The University of Iowa Animal Care and Use Committee.

## Author Contributions

RT conceptualized experiments, collected and analyzed data, and wrote the manuscript. AW conceptualized experiments, collected and analyzed data, and revised the manuscript. GW collected data. AC, BD, and JW conceptualized experiments and revised the manuscript. All authors contributed to the article and approved the submitted version.

## Conflict of Interest

The authors declare that the research was conducted in the absence of any commercial or financial relationships that could be construed as a potential conflict of interest.

## Publisher’s Note

All claims expressed in this article are solely those of the authors and do not necessarily represent those of their affiliated organizations, or those of the publisher, the editors and the reviewers. Any product that may be evaluated in this article, or claim that may be made by its manufacturer, is not guaranteed or endorsed by the publisher.
