# Supplementary material for: Post-acquisition CO2 Inhalation Enhances Fear Memory and Depends on ASIC1A
Source: Front Behav Neurosci. 2021 Oct 29;15:767426. doi: 10.3389/fnbeh.2021.767426 (PMC8585996; doi:10.3389/fnbeh.2021.767426)
Supplement: Supplementary file 1 [file Table_1.pdf]

Table S1: Absolute values for freezing. Non-normalized freezing values for all experimental groups.

Figure	Group	Time freezing in sec (mean ± SEM)
1B	No Shock, Air	46.5 ± 12.0
	No Shock, 10% CO_2	36.0 ± 6.1
	Shock, Air	250.9 ± 28.6
	Shock, 10% CO_2	263.9 ± 20.1
1C	No Shock, Air	12.7 ± 5.5
	No Shock, 10% CO_2	9.8 ± 3.7
	Shock, Air	113 ± 9.8
	Shock, 10% CO_2	155.8 ± 3.3
1E	Control	260.1 ± 23.5
	Restraint	268 ± 20.3
1F	Control	111.6 ± 11.7
	Restraint	122.8 ± 8.58
2B	Air	257.9 ± 19.9
	10% CO_2	302.3 ± 14.7
2C	Air	114.2 ± 7.0
	10% CO_2	148.6 ± 3.7
2E	Air	355.9 ± 11.8

	10% CO_2	333.6 ± 24
2F	Air	147 ± 5.2
	10% CO_2	129.6 ± 10.2
2H	Air	405.2 ± 11.4
	10% CO_2	371.3 ± 12.6
2I	Air	157.7 ± 5.1
	10% CO_2	152.4 ± 3.6
3B	Air	108.6 ± 6.1
	10% CO_2	108.5 ± 6.5
3C	Air	100.3 ± 7.1
	10% CO_2	155.3 ± 13.9
4B	Air	180.8 ± 27.9
	10% CO_2	157.4 ± 28.6
4C	Air	61.1 ± 9.3
	10% CO_2	62.0 ± 6.3
4E	Air	83.6 ± 7.3
	10% CO_2	80.4 ± 16.5
4F	Air	70.7 ± 10.7
	10% CO_2	68.4 ± 17.1
4H	Air	63.6 ± 10.2

	10% CO_2	55.1 ± 8.7
4I	Air	77.1 ± 14.7
	10% CO_2	65.7 ± 11.6
